# 

**Published:** 1890-01

**Authors:** 


### INDEX TO VOLUME XLIV.

### COMMUNICATIONS.

A Case in Hand.................... 345
A Consideration of Dental Patents .......................338
Address Dr. W. H. Sedgwick 589
Address of Dr. J. B. Patrick Before the Southern Dental Association............... 431
Address, President's.............. 180
Address to the Section of Dental and Oral Surgery....... 451
Affections Liable to Occur to the Alveolar Process........ 218
Anæsthesia ........................... 479
Antrum, Diseases of the....... 5
Antrum, Diseases of the......... 269
Chloroform, Death from........ 16
Clinical Observations on Operations for Congenital Clefts in Gums, Lips and Palate in Early Infancy... 456
Cure of Cleft Palate by a Double Flap Operation and Closure with the Buried Tendon Suture..... 577
Dental Education — Office Pupilage ...................... 57
Dentistry, Evolution in 61, 123, 191, 213, 265, 375, 446
Dentistry, Operative.............. 437
Dentistry, Operative............. 473
Dentists, The Wealth of......... 170
Do We, in our Practice, do the best in Our Power for Our Patients?....................... 582
Does Our Profession Confer all the Benefits it Should? 358
Hands, Chapped.................... 21
Hereditary Character of Deformities...................... 369
How to Stand....................... 127
Hypnotism, Dangers of......... 531
Imagination........................ 161
Importance of Dentistry to the Physician ...................... 535
Intimate Diagnosis of Lesions affecting the Teeth......... 598
Inventions and New Things... 165
Invitation to the Tenth Medical Congress.................. 363
Is the Six Year Molar less Perfect than the Other Teeth ........................... 67
Magnesic Sulphas................. 197
Maxillæ, Fractures of the...... 528
New Italian Review on Hygiene......................... 12
Non-Metallic Plastic Materials for Filling Teeth............ 175
Ohio State Dental Society—Discussion of Dr. F. W. Sage's Paper.................. 606
Oil of Birch and Oil of Wintergreen........................ 381
Oral Secretions, Changes of the 117
Professional Ethics and Honor 183
Reduction of Fracture of Superior Maxillary............ 357
Rhino-Plasty ....................... 221
That Barbaric "Key"............ 533
The Necessary Peroxide of Hydrogen ...................... 454
The Physical Change Produced by Decay ...................... 17
The Physiology and Pathology of the Peridentium........ 441
The Teeth and Oral Cavity of Pregnant Women............ 109
The Value of Illustration in the Lecture Room........... 379
Thirty-Fifth Annual Meeting of the Michigan State Dental Association......... 317
Tooth Pulp Functions of........ 69
Tropho-Neurosis of the Oral Cavity with Especial Reference to Syphilitic Necrosis........................... 421
Tumors ............................... 13

### PROCEEDINGS.

Annual Meeting of the Northern Ohio Dental Association ............................. 308
Association, The Dental Protective......................... 398

Association, Missouri State Dental ........................ 405
Constitution of the National Association of Dental Examiners ........................ 545
Dental Ethics ........................ 386
International Congress ........................ 309
National Association of Dental Faculties ........................ 548
The Forty-Sixth Annual Meeting of the Mississippi Valley Association of Dental Surgeons ........................ 225
The National Association of Dental Examiners ........................ 536
The Thirtieth Annual Meeting of the American Dental Association ........................ 481
Thirty-Fifth Annual Meeting of the Michigan State Dental Association ........................ 386
Treatment of Second Stage of Alveolar Abscess ........................ 392

SELECTIONS.

A Case of Congenital Lockjaw 564
Administration of Anæsthesia 36
A Bungling Chemist's Discovery—Test for Blood ........................ 48
Alcohol and Tea ........................ 588
Antiseptic Properties of Corrosive Sublimate in Minimal Quantities ........................ 79
Antiseptics Among the Ancient Greeks ........................ 565
A Leech in the Larynx ........................ 136
An Infant Prodigy ........................ 566
A Simple Remedy for Thrush and Sordes ........................ 138
A National Department of Public Health ........................ 199
A Paste That will Adhere to Anything ........................ 203
American Medical Editor's Association ........................ 242
A Study of the Relationship Between the Eruption of the Permanent Teeth and the Ailments of Late Childhood ........................ 275
About Platinum ........................ 301
Another Warning to Landlords 306
Anæsthesia—Nitrous Oxide... 519
Beer Compared with Other Alcoholics ........................ 45
Bromide of Lithia ........................ 202
Certain Effects of Tobacco Smoking. Colored Spectacles ........................ 96
Chloroform Caution ........................ 614
Cocaine. Bacteriology ........................ 103
Chronic Syphilitic Salivations 304
Chicago Dental Society ........................ 305
Dentistry, Evolution in ........................ 568
Department of Hygiene at the University of Pennsylvania ........................ 34
Death from Nitrous Oxide..... 614
Death After Ether Anæsthesia 35
Do not Waste Alcohol ........................ 37
Digestibility of Boiled Milk... 77
Decomposition of Iodoform Solutions. Bacillus of Tetanus ........................ 93
Dangers of Celluloid ........................ 132
Death in Soothing Syrup ........................ 202
Doctors and Dentists with American Diplomas ........................ 240
Do Heads Grow with Advanced Age? ........................ 241
Dental College Commencements ........................ 243
Dental Laws of North Dakota 295
Dangers of Hypnotism ........................ 303
Disinfecting Means and Methods ........................ 557
Dentition ........................ 514
Disinfecting Power of Peroxide of Hydrogen ........................ 517
Effects of Heat and Cold—How to Kill Bacteria ........................ 567
Extract from Clinical Lecture, Primary Dentition ........................ 28
Excision of the Facial Bones.. 130
Experimental Investigations Concerning Suppuration.. 513
Female Physicians in Earlier Times ........................ 616
For Trigeminal Neuralgia..... 563
Facial Neuralgia and Allied Neurosis ........................ 201
Facial and Thoracic Deformities Incident to Obstruction by Adenoid Hypertrophy in the Naso-Pharynx ........................ 551
Gastric Digestion ........................ 87
Guy's Hospital Dental School. 242
Hygiene and Sunday ........................ 81

Hyderabad Chloroform Commission ........................ 234
Integrity in Medical Colleges. 76
Intertrigo. Four at a Time... 92
International Medical Congress ............................ 146
Letter from Berlin................ 38
Local Anæsthesia by Cocaine.. 134
Mouth Washes. Food and Sleep ......................... .. 82
Medical Congress ................. 88
Miscellaneous Items..........94, 95
Mania Following Ether......... 567
Miscellaneous Items............. 102
Mouth-Breathing and the Teeth ............................. 129
Menthol in Acute Rhinitis, etc 133
Mind Blindness..................... 135
Miscellaneous Items........140, 143
Nitrous Oxide Gas — The Phonograph as a Reporter 46
Notes and Comments—A Curious Hypnotic Test........... 239
Nerve Restoration................. 516
New Method for Vulcanizing Rubber Plates................. 562
Operation and Prognosis of Lingual Cancer. A Belgian View of Medical Advancement ..................... 91
Practical Notes..................... 31
Pure Air in Churches............ 74
Peroxide of Hydrogen as Antiseptic and Disinfectant.. 83
Preventive Therapeutics. Mercuric Chloride. Sterilized Lint. Local Anæsthesia .......................... 85
Plastic Surgery...................... 304
Powerful Antiseptic.............. 464
Remedy for Tender Feet...... 588
Removal of Breast During Hypnotic Sleep................ 561
Rules for Good Health—Eating Before Sleeping......... 97
Remedy for La Grippe........... 563
Solvents for Camphor............ 616
Some Notes on the Nails........ 554
Some Discussions on Tuberculosis, etc......................... 22
Source of Colors................... 44
Statistics of Breathing—A new Cure for Toothache......... 47
Statistics of Breathing........... 139
The New Hypnotic, Sulphonal 41
The County Dental Society of Philadelphia.................. 615
The Scientific Uses of Very High Towers.................. 25
The Native Egyptian as a Subject of Surgical Operation.............................. 35
The Use of Antiseptics in Dentistry........................ 43
The Value of the Examination of the Teeth in Anthropological Research............ 609
Tenth International Medical Congress, Berlin. 1890...... 49
The Anatomy of the Future... 71
The Graphic Arts in Medicine 73
The Drink Question in France 78
Thumb Sucking.................... 84
Treatment of Hemorrhage after Extraction of Teeth. Diet of Strong Men.......... 86
The Chinese Way. Headache 89
The Practical Physician. Miscellaneous Items....98, 99
The Normal Diet. Number of Dentists in Germany... 100
Transmission of Scarlatina. Treatment of Tetanus...... 101
Treatment of a Cold. Association News...................... 104
The Action of Microbial Products on Microbes and on the Organism................. 559
The Red Line Along the Gums ........................... 137
The Exciting Cause of Tetanus 144
The Regulation of Sleep........ 200
The Circulation of the Blood .. 464
Thymol as an Intestinal Antiseptic ........................... 510
The Relation of Mastication to Physical Development. 510
The Wearing Away of Teeth.. 512
The International Congress from an English Standpoint ...... .................... 518
Virchow and the Darwinian Theory........................ 74
Women Physicians in India... 615
Work Kills no Man.............. 78
Wounds of Inferior Maxillary Bone.................................. 90
World-Wide Brotherhood...... 553

### COMMENCEMENTS.

Boston Dental College........... 410
College of Dental Surgery of the University of Michigan .............................. 409
Harvard University—Dental Department ...................... 410
Northwestern University—Dental Department......... 409
University of Pennsylvania—Dental Department......... 408

### CORRESPONDENCE.

Atlanta, Georgia.................. 411
Battle Creek, Michigan......... 412
Letter from Rome, Italy........ 150
Letter fromBudapest,Hungary 151
Letter from London, England 154
Letter from Geo. A. Mills, New York...................... 204
Northern Indiana Dental Society .......................... 51
Trouble in the Camp............. 253
The Tenth International Medical Congress.................. 465

### EDITORIAL.

An International Dental Congress ......................... 105
American Medical Association 260
A New Medical Dictionary.... 260
American Medical Association 311
American Dental Association.. 312
Association of Dental College Faculties ...................... 313
A Dental Gathering—1893..... 365
American Dental Association. 520
Are Dentists Honest? ........... 575
Bibliographical.................... 367
Bibliographical.................... 522
Bibliographical.................... 621
Change of Time..................... 521
Contagion Vivum................. 523
Dental Society Work............ 257
Dental Anatomy................... 261
Dr. S. B. Hartman............... 622
Dr. Walter Rivington........... 618
Editorial Change.................. 106
Experiments with the Electro-Deposit Plate................... 210
Educational—The Dental Colleges.......................... 470
Georgia State Dental Society.. 314
Glossary of Terms Applied in Electrical Science........... 617
Honorable Mention.............. 619
Is It a Fraud?...................... 157
International Medical Congress—Dental Section...... 158
International Medical Congress........................... 255
Iowa State Dental Association 259
In Memoriam....................... 420
National Association of Dental Faculties.................. 56
Northern Ohio Dental Association.......................... 264
National Association of Dental Examiners................ 313
Notices ............................. 367
New Arrangement, Editorially 572
Odontological Society of Cincinnati ........................ 52
Obituary, Wm. M. Hunter..... 54
Obituary, Wm. A. Pease........ 55
Obituary, W. T. King............ 212
Obituary, L. B. Welch........... 315
Obituary, C. Atkinson........... 368
Obituary, H. Judd................ 419
Ohio State Dental Society...... 469
Ohio State Dental Society...... 521
Ohio State Meeting Jottings... 620
Obituary, W. H. Woodward... 524
Professor C. L. Ford's Questions.......................... 107
Percentage of Graduates........ 621
Personal ............................ 468
Personal—Removal.............. 472
Prof. W. D. Miller—A Reception............................ 572
Removal ............................ 52
Student's Society.................. 53
Seventh District Dental Society—Ohio ...................... 254
Should Physicians be Illiterate 258
Southern Dental Association.. 417
State Board of Dental Examiners ........................... 469
The Dental Protective Association ........................ 156
The Chase Metallic Combination Plate...................... 211
Transactions of the American Dental Association......... 212
The Metric System............... 574
The Dental Protective Association ........................ 262
What About It?.................. 211
What a Dentist has Done...... 619

### NOTICES.

American Medical Association 205
International Medical Congress of 1890................... 207
Programme of the Dental Section.............................. 207
Report of the Committee on Foreign Transportation... 208
Tenth International Medical Congress — Preliminary Programme of the Dental Section.............................. 209

### EDITORIAL.

### Removal.

Professor J. E. Cravens, of Indianapolis, has associated himself in dental practice with Dr. E. A. Bogue, of No. 73 Boulevard Hausmann, Paris, France.

Dr. Cravens has long been one of the leading members of the profession in Indiana, one who has for many years exercised a great influence on behalf of the profession in that State. He was one of the most active in the establishment of the Indiana Dental College, and in bringing it to its present honorable and useful status. He was indefatigable in his efforts for its upbuilding and improvement, and he leaves it as a monument to his effort and wise guidance. His removal is a loss to the profession of Indiana, and to others as well. The profession can ill afford to spare such men from its ranks.

There is some gratification, however, in the fact of such men going abroad as representatives of the profession in this country. It will not there surely suffer at his hands.

Dr. Bogue, who has for many years had an established business in Paris, has been very fortunate in this alliance, and in it he acted with a full knowledge of what he was doing, for he had Dr. Cravens associated with him for about a year on a former occasion. Long and happily may they enjoy the fruits of this association, is the wish of not only the editor but of all who know Drs. Bogue and Cravens.

---

Annual programme of the Odontological Society of Cincinnati, for 1889-90. (Incorporated February 23d, 1886). Regular meetings on the fourth Tuesday of each month.

OFFICERS.

Dr. M. H. Fletcher, President; Dr. Grant Molyneaux, Vice-President; Dr. W. M. Williams, Secretary.

BOARD OF TRUSTEES.

Dr. D. W. Clancey, Chairman; Dr. H. L. Moore, Treasurer; Dr. J. S. Cassidy, Dr. W. D. Phillips, Dr. H. A. Smith.

COMMITTEE ON PROGRAMME.

Dr. M. H. Fletcher, Chairman ; Dr. D. W. Clancey, Dr. W. M. Williams.

PROGRAMME.

Voluntary papers, clinics and incidents of office practice at each meeting.

October 26. "Pyorrhœa Alveolaris," Dr. D. W. Clancey.

November 26. "Dental Pupilage,"................Dr. J. Taft.

December 24. "Duty and Its Relations to Association Work.".........................................-Dr. H. L. Moore.

January 28. "Quacks.".................Dr. W. M. Williams.

February 25. "Imagination."..........Dr. J. R. Callahan.

March 25. "Intimate Diagnosis of Lesions Affecting the Teeth."..........................................Dr. Frank W. Sage.

April 22. "Etiology of Dental Caries."...Dr. H. A. Smith.
(Election of officers at this meeting).

May 27. "Vulcanite Plates."......Dr. Grant Molyneaux.

"Diseases of the Teeth and their Effects on Constitutional Conditions."...........................................Dr. C. H. Martin.

June 24. "Histology of Repair in Animal Tissue."............
.......................................................Dr. M. H. Fletcher,

Society adjourns to October, 1890.

### Students' Society.

### Obituary.

NATIONAL ASSOCIATION OF DENTAL FACULTIES.—*Special notice.* The resignation and removal to Paris, France, of Dr. J. E. Cravens, has made necessary the appointment of a secretary to fill his position. On the recommendation of the Executive Committee, I have appointed Dr. John S. Marshall of Chicago.

All communications requiring the attention of this organization should hereafter be sent to his address, cor. Michigan Ave. and Jackson Sts., Chicago, Ill. JAMES TRUMAN,

Pres. N. A. of D. F.

### CONTENTS.

### COMMUNICATIONS.

Diseases of the Antrum.......... 5

New Italian Review on Hygiene.......... 12

Tumors.......... 13

Death from Chloroform.......... 16

The Physical Changes Produced by Decay.......... 17

Chapped Hands.......... 21

### SELECTIONS.

Some Discussions on Tuberculosis, etc.......... 22

The Scientific Uses of Very High Towers.......... 25

Extract from Clinical Lecture, Primary Dentition.......... 28

Practical Notes.......... 31

Department of Hygiene at the University of Pennsylvania.......... 34

The Native Egyptian as a Subject of Surgical Operation.......... 35

Death after Ether Anæsthesia.......... 35

Administration of Anæsthetics.......... 36

Do Not Waste Alcohol.......... 37

Letter from Berlin.......... 38

The New Hypnotic-Sulphonal.......... 41

The Use of Antiseptics in Dentistry.......... 43

Source of Colors.......... 44

Beer Compared with other Alcoholics.......... 45

Nitrous Oxide Gas—The Phonograph as a Reporter.......... 46

Statistics of Breathing—A New Cure for Toothache.......... 47

A Bungling Chemist's Discovery—Test for Blood.......... 48

Tenth International Medical Congress, Berlin, 1890.......... 49

### CORRESPONDENCE.

Northern Indiana Dental Society.......... 51

### EDITORIAL.

Removal.......... 52

Odontological Society of Cincinnati.......... 52

Students' Society.......... 53

Dr. William M. Hunter.......... 54

Obituary.......... 55

National Association of Dental Faculties.......... 56

---

### THE DENTAL REGISTER,

A MONTHLY MAGAZINE DEVOTED TO THE INTERESTS OF THE

**DENTAL PROFESSION.**

*Terms Reduced to $2.00 Per Year. Single Copies, 25 Cents.*

**EDITED BY PROF. J. TAFT, D.D.S.**

—AND—

**Published by SAM'L A. CROCKER & CO., Ohio Dental and Surgical Depot.**

The volume commences in January and closes in December.

The REGISTER being issued monthly is always fresh, therefore Indispensable to the practitioner who desires to keep posted up with the new inventions and improvements which are daily developed. Neither expense nor effort will be spared to give value and variety to its contents. Articles will be illustrated with well executed engravings whenever they will add to the interest or assist to explanation.

Its extensive circulation and frequency of issue make it one of the BEST DENTAL ADVERTISING MEDIUMS in the United States.

All subscriptions must begin with January or July.

Local or special notices, five lines or less, will be inserted for $3 each insertion; over five lines, 50 cts. per line.

All advertising bills payable in advance, or at furthest, after the issue of the first number containing the advertisement. All transient advertisements to be paid for in advance.

☞CONTRIBUTIONS SOLICITED.

Exclusively Editorial Communications should be addressed to the Editor.

The latest number of the REGISTER may be ordered with or without other goods at any time, and all letters should be addressed to

